# Tuning the band gap, optical, mechanical, and electrical features of a bio-blend by Cr_2_O_3_/V_2_O_5_ nanofillers for optoelectronics and energy applications

**DOI:** 10.1038/s41598-024-62643-6

**Published:** 2024-05-31

**Authors:** Tarek I. Alanazi, Raghad A. Alenazi, Adel M. El Sayed

**Affiliations:** 1 Department of Physics, College of Science, Northern Border University, 73222 Arar, Saudi Arabia; 2 Physics Department, Faculty of Science, Fayoum University, El-Fayoum, 63514 Egypt

**Keywords:** Cr_2_O_3_/V_2_O_5_ nanoparticles, CMC/PEG biopolymer blend, Band gap engineering, Energy density, Dielectric modulus, Stress–strain, Materials science, Nanoscience and technology, Optics and photonics, Physics

## Abstract

This work presents a facile approach for controlling the optical and electrical parameters of a biopolymeric matrix for optoelectronics. Vanadium oxide (V_2_O_5_) and chromium oxide (Cr_2_O_3_) nanoparticles (NPs) were prepared and incorporated into the carboxymethylcellulose/polyethylene glycol (CMC/PEG) blend by simple chemical techniques. Transmission electron microscopy (HR-TEM), and X-ray diffraction (XRD) data showed that V_2_O_5_ and Cr_2_O_3_ exhibited spherical shapes with sizes in the range of 40–50 nm and 10–20 nm, respectively. In addition, the blend's degree of crystallinity was sensitive to the V_2_O_5_ and Cr_2_O_3_ doping ratios. The scanning electron microscopy (FE-SEM) and the elemental chemical analysis (EDAX) used to study the filler distribution inside the blend, and confirmed the existence of both V and Cr in the matrix. Fourier transform infrared (FTIR) spectroscopy showed that the dopants significantly affected the blend reactive (C–O–C, OH, and C=O) groups. The stress–strain curves illustrated the reinforcing effect of the dopants up to 1.0 $\text{wt\%}$ Cr_2_O_3_/V. The transmittance and absorption index spectra in the visible-IR wavelengths decreased with increasing filler content. Utilizing Tauc's relation and (optical) dielectric loss, the direct (indirect) band gap narrowed from 5.6 (4.5) eV to 4.7 (3.05) eV at 1.0  $\text{wt\%}$ Cr_2_O_3_/V. All films have an index of refraction in the range of 1.93–2.17. AC conductivity was improved with increasing filler content and temperature. The energy density at 50 °C is in the range of 1–3 J/m^3^. The influence of V_2_O_5_ and Cr_2_O_3_ content on the optical conductivity, dielectric constant, loss, and dielectric modulus of CMC/PEG was reported. These enhancements in electrical and optical properties, along with the potential for band gap engineering, offer promising prospects for advanced applications in optoelectronics and energy-related fields.

## Introduction

Biopolymer nanocomposites (BPNC) are made by incorporating a tiny amount of inorganic nanofillers into a biopolymer matrix. Besides the simplicity in fabrication and handling, the key factors influencing the BPNC' properties for the targeted applications include the nature of the polymer matrix, filler composition, size, and quantity, and the homogeneity degree inside the BPNC^1,2^. Blending at least two biopolymers is a simple and cost-effective approach for obtaining new materials with desirable characteristics that can't be obtained from a polymer^3^. Nano-sized materials in the form of nanoparticles (NPs) exhibit unique features owing to their reactivity, and huge surface area^4^. Loading NPs induces modifications in the blend's electronic structure and energy gap. Therefore, the resulted nanocomposites can be used as multifunctional materials for advanced technological applications such as optoelectronic devices, solar cells, light-emitting (organic) diodes, memory devices, image sensors, electrochemical, and energy-related uses^1,3,5^.

The chemical versatility of carboxymethylcellulose (CMC, also known as cellulose gum) arises because it is a basic cellulose derivative that consists of Na carboxymethyl (CH_2_COONa) attached to a polysaccharide (cellulose) backbone^4,6^. CMC is semi-crystalline ionic-type cellulose ether, an environmentally friendly, biodegradable, and inexpensive polymer extract from the fibrous tissue of plants, fruit, and vegetable-based wastes^7,8^. Owing to its exceptional viscosity, biocompatibility, flocculating property, high water absorption, thermal gelatinization, transparency, and ability to form a continuous medium, CMC can form flexible and strong films^9,10^. These features made CMC the best choice as an adhesive, thickening agent, and stabilizer for improving the processing ability of textiles, cosmetics, paper, foodstuffs, creams, lotions, toothpaste compositions, and drug delivery^10–15^. On the other side, poly(ethylene glycol), or PEG, exhibits low toxicity, and excellent water solubility^16^. In the blending process, PEG is usually used as a pores-forming agent to enhance the free volume in the matrix and to allow the chain segments more freedom to move and rotate^17,18^.

The production and use of CMC-based edible films have become essential for food recycling and sustainability^7^. Salem et al.^19^ reported that adding 10 $\text{wt\%}$ polypyrrole to CMC raised the blend's refractive index (*n*) from 2.11 to 2.64 and greatly decreased the band gap (*E*_g_). This made the blend ideal for advanced optoelectronics. With the addition of starch and cellulose nanocrystals, the mechanical features and viscoelastic behavior of CMC can also be changed to suit farming and packaging needs^20^. A previous study reported that blending PEG with PVA improved the refractive index of anti-reflective coatings^21^. El Askary et al.^6^ improved the optical characteristics of CMC/PVA by forming Er_2_O_3_ NPs inside the blend utilizing the laser ablation technique. Menazea et al.^12^ fabricated MoO_3_/PEO/CMC nanocomposites by a hydrothermal method and solution casting. According to Kodsangma et al.^14^, the ionic interaction and physical cross-linking of OH with Zn^+^ in ZnO/CMC/thermoplastic starch improved its thermal, mechanical, and water resistance. The optical parameters of PVA/CMC/PEG modified with Zn_0.9_Cu_0.1_S and Zn_0.95_V_0.05_S were reported in Ref.^9,16^. The energy density and dielectric moduli of PVC/PEG changed irregularly with increasing CeO_2_ NPs content^18^. According to El-naggar et al.^22^, PVP/CMC mixed with 5.0 $\text{wt\%}$ hydrogen titanate nanotubes/tetramethylammonium iodide could be used in optoelectronics. Al-Muntaser et al.^13,23^ studied the band gap structure, dielectric moduli, and impedance of a CMC/PVA/PVP mixed with ZnO or TiO_2_ NPs. Farea et al.^24^ improved the semiconducting properties of PVA/CMC by introducing CdO NPs.

Among the transition metal oxides as nanofillers, the rhombohedra chromium oxide (Cr_2_O_3_) is a stable material with *p*-type, wide *E*_g_ (3.1–3.4 eV), mobility of 0.14 cm^2^ V^−1^s^−1^, and high energy density^10^. Therefore, Cr_2_O_3_ is suitable for photonics, optical storage devices, nanolasers, and also as a cathode material^25,26^. Additionally, the limited size and high density of corner/edge sites give the nano-sized vanadium oxides a distinctive geometry^27^. Vanadium pentoxide (V_2_O_5_) exhibits a V^5+^ oxidation state and acts as a semiconductor of *n*-type, a relatively narrow *E*_g_ (1.9–2.3 eV) and exceptional thermoelectric and electrochromic features. In addition, it has been used effectively in diverse fields such as gas sensing, photocatalysis, energy storage, and supercapacitors^28,29^. The simultaneous doping with two transition metal oxides can provide composites with interesting biological features for biomedical or clinical applications^30^. El-Morsy et al.^2,11^ investigated the opto-electrical features of CdO/Al_2_O_3_/CMC and Cr_2_O_3_/TiO_2_/CMC composites. Gaabour^31^ studied the dielectric characteristics of Cr_2_O_3_/CMC/PEO nanocomposites. Hamza and Habeeb loaded SiO_2_/Cr_2_O_3_/PVA/CMC nanocomposites and proposed their use as antimicrobial films for the food industry^10^. Menazea et al.^27^ reported improved antibacterial efficacy for the chitosan/PVA with increasing V_2_O_5_ NPs content. Awwad et al.^32^ reported that V_2_O_5_ NPs created by laser ablation inside PEO/chitosan matrix changed the band gap and conductivity of the blend in a non-monotonic behavior with increasing ablation time.

The literature survey revealed that no complete reports are found on Cr_2_O_3_/V_2_O_5_/CMC/PEG bio-nanocomposites. Our focus and interests are to combine the unique features of V_2_O_5_, Cr_2_O_3_, and CMC/PEG in composites, and explore the influence of simultaneous doping with V_2_O_5_ and Cr_2_O_3_ on the structural features, interactions, and physical characteristics of the CMC/PEG blend. The results revealed that Cr_2_O_3_/V_2_O_5_ can tune and engineer the blend structure, mechanical, optical, and electrical properties. This encourages the use of prepared flexible nanocomposites for some optoelectronic and energy-storing devices.

## Experimental section

### Chemicals, methodology, and free-standing composite film fabrication

Chromium (III) acetate [Cr(C_2_H_3_O_2_)_3_, 229 g/mol, Med Chem Express, USA], vanadium (V) oxide [V_2_O_5_, 181.18 g/mol, Merck], HCl, oxalic acid [C_2_H_2_O_4_·2H_2_O, 126 g/mol], carboxymethylcellulose [CMC, (OCH_2_COONa)_y_C_6_H_7_O_2_(OH)_x_]_n_, (y + x = 3), 0.29 × 10^6^ g/mol, El Nasr Pharmaceutical Chemicals Co., Egypt] and polyethylene glycol [PEG, H(OCH_2_CH_2_)_n_OH, 6000 g/mol, DOP Organic Kimya, Konya, Turkey]. Distilled water was used as a solvent.

V_2_O_5_ (1.1 g) was dissolved in HCl (30 ml) by stirring for 30 min. The obtained solution was heat-treated at 100 °C for 6 h. The green product was then dissolved in ethanol, dried, and then annealed at 400 °C for 1 h to obtain V_2_O_5_ NP. Cr(C_2_H_3_O_2_)_3_ (1.15 g) and oxalic acid (0.63 g) were dissolved in pure water (25 mL) by stirring for 1.0 h/50 °C. The sample was put in a furnace at 100 °C to dry (remove the excess water). Then it was subjected to a thermal decomposition at 400 °C/1.0 h to obtain solid Cr_2_O_3_ NPs.

CMC (0.9 g) was dissolved in distilled water (50 mL) by stirred at 85 °C. PEG (0.1 g) was also dissolved in distilled water (10 mL) at RT. These dissolutions took 1.0 h to obtain transparent and clear CMC and PEG solutions. Then the two solutions were mixed and stirred for 1.0 h, at RT. The nanocomposite solutions were prepared following the same procedures with the addition of 0.5 $\text{wt\%}$ V_2_O_5_, 0.5 $\text{wt\%}$ Cr_2_O_3_/0.5 $\text{wt\%}$ V_2_O_5_, 1.0 $\text{wt\%}$ Cr_2_O_3_/0.5 $\text{wt\%}$ V_2_O_5_, and 2.0 $\text{wt\%}$ Cr_2_O_3_/0.5 $\text{wt\%}$ V_2_O_5_ to the blend solution. These fillers' contents were calculated using the relation: $x \left(\text{wt\%}\right)=\frac{{m}_{\text{f}} }{{m}_{\text{f}} + 1} \times 100\%$, where ${m}_{\text{f}}$ is the mass of the filler (V_2_O_5_ or Cr_2_O_3_/V_2_O_5_) and the "1" is the mass of the polymers. The uniformly mixed blend and nanocomposite solutions were poured into Petri dishes (glass) and left for more than 24 h to dry in a controlled environment at 40–45 °C.

### Devices and characterization

The structure and morphology of the fillers (V_2_O_5_, Cr_2_O_3_) were performed using field emission SEM, model Carl ZEISS (Sigma 500 VP), and TEM (JEM 2100, Jeol, Japan). A diffractometer (PANalytical/X’Pert/PRO) with copper K_*α*_ lines of wavelength *λ* = 0.154 nm, and step 0.02° was used to study the crystal and phases of V_2_O_5_ and Cr_2_O_3_, CMC/PEG and BPNC films. The films' morphology explored using FE-SEM. FTIR spectra were collected in 4000–400 ${\text{cm}}^{-1}$ wavenumbers, using a Bruker/vertex70 spectrophotometer. Stress vs. strain (i.e., tensile testing) was obtained utilizing a testing machine of ZwickRoell, Z010 TN-Germany, operated at 1 × 10^3^ N. The toughness was measured from the integrated area under the obtained curves using the Origin Pro software. The films of CMC/PEG and Cr_2_O_3_/V_2_O_5_/CMC/PEG were cut in a specific shape (length and width of 8 cm and 1.5 cm, respectively), where the test was done three times for each sample at a speed of 50 mm/min. UV–Vis-NIR transmittance (T%) and absorption (Abs.) in 200–1400 nm wavelengths, were recorded by a Shimadzu spectrophotometer (UV-3600/UV/Vis/NIR). An accurate (± 0.001 mm) digital micrometer evaluated the sample thickness. All of these measurements were performed at RT. The dielectric features and parameters were considered using a broad-band dielectric spectroscopy (Novocontrol turnkey 40) System, across 10^−1^ Hz–10 MHz frequencies at different three temperatures (30, 50, and 70 °C).

## Results and discussion

### Morphology and structure of the sol–gel-derived NPs

**Figure 1:**
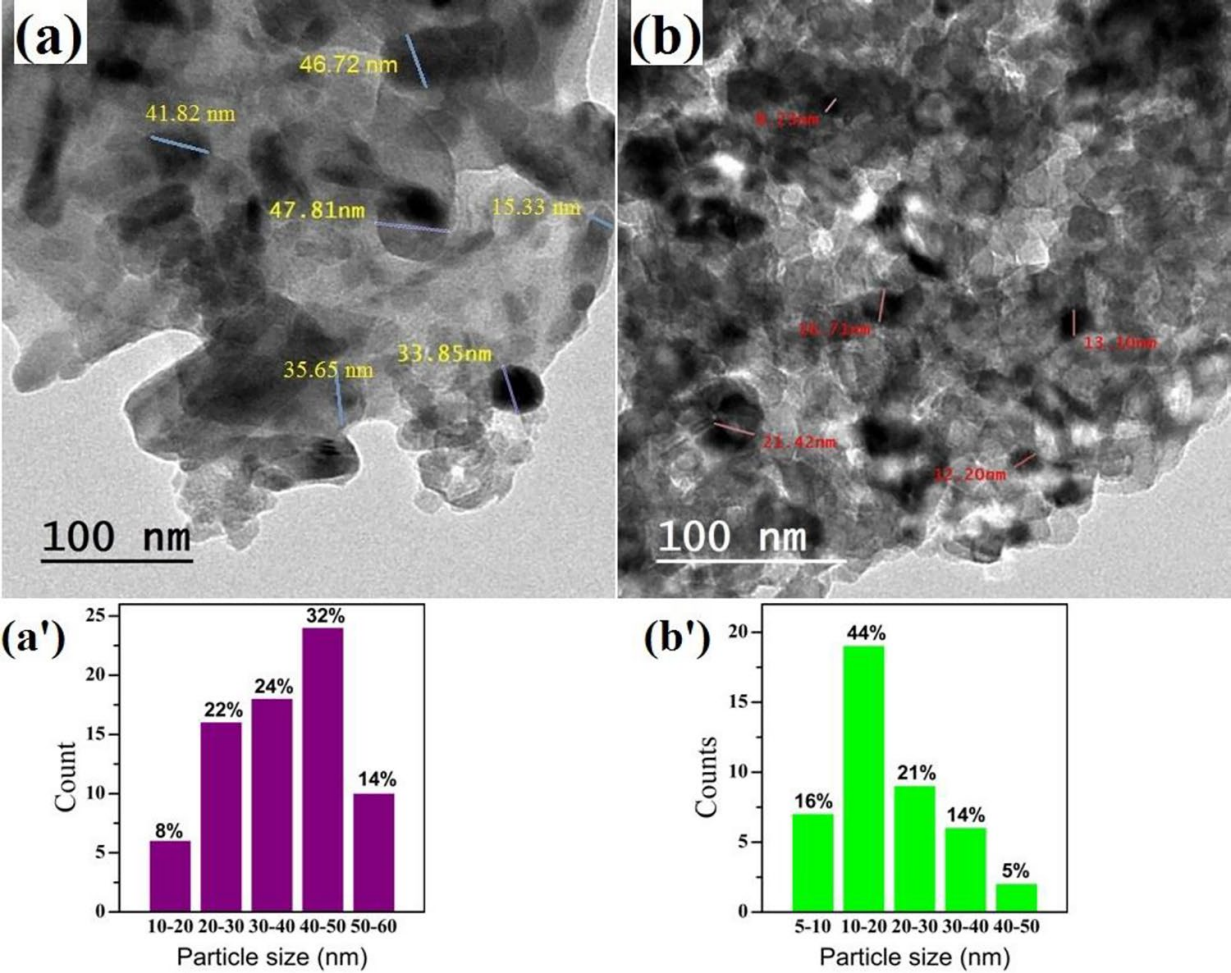
HR-TEM images for (**a**) V_2_O_5_, (**b**) Cr_2_O_3_ NPs. (**a′**,**b′**) show the corresponding histogram particle size distribution.

The morphology of the sol–gel-prepared V_2_O_5_ and Cr_2_O_3_ was checked by HR-TEM, as shown in Fig. 1a,b, and also FE-SEM, Supplementary Fig. S1a,b. See the supporting materials file. The powders are composed of NPs, and the size of V_2_O_5_ (Fig. 1a) is in the range of ~ 40–50 nm. Also, Cr_2_O_3_ exhibits more spherical particles, 10–20 nm in size. Figure 1a′,b′ shows a histogram of the particle size distribution of both V_2_O_5_ and Cr_2_O_3_, respectively. Supplementary Figure S1a,b also shows a particle morphology for the powders. As observed in Fig. 1a,b, V_2_O_5_ NPs agglomerate and appear with a larger grain size. This NPs adhesion is due to covalent or metallic bonds which form micro-sized structures^33^.

**Figure 2:**
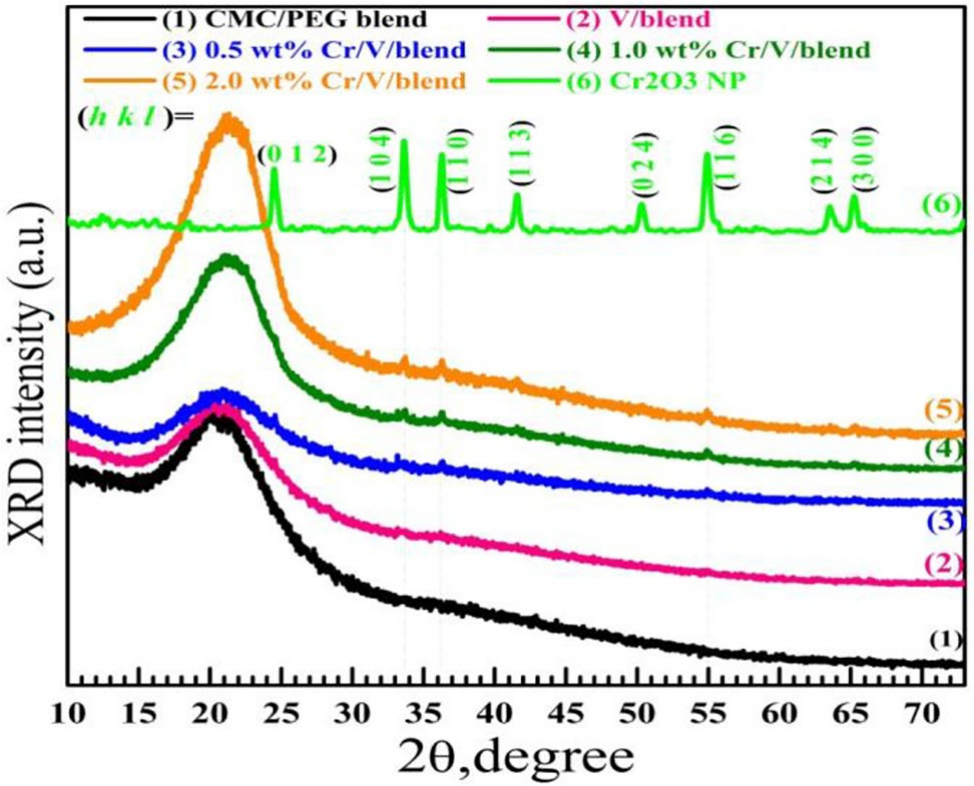
XRD charts of CMC/PEG blend, V_2_O_2_/blend, Cr_2_O_3_/V_2_O_5_/blend and Cr_2_O_3_ NP.

Figure 2 shows the XRD spectrum of Cr_2_O_3_. Well-defined diffraction peaks appear at $2\uptheta$ = 24.45°, 33.55°, 36.25°, 41.48°, 50.29°, 54.85°, 63.62°, and 65.22°, which correspond to Bragg reflections of (*hkl*) = (012), (104), (110), (113), (024), (116), (214), and (300), respectively. This chart indicates the formation of Cr_2_O_3_ with rhombohedra structure, $R\overline{3 }c$ SG, and parameters (lattice): *a* = 4.957 Ǻ & *c* = 13.584 Ǻ, is coincident with the data of JCPDS no.: 84-1616. The Sherrer's formula: $D= \frac{K \times {\lambda }_{Cu}}{FWHM.\text{cos}\theta }$, where ${\lambda }_{Cu}$ = 0.154 nm, *K* is the Sherrer's constant ($0.9$), and *FWHM* of the peak is the width at half maximum intensity, was used for the average crystallite size *D* determination, which was found in the order of = 25.36 nm. While FE-SEM measures the distances between the grains (grain or particle size), XRD calculates *D*, which may represent a part of the grain^34^. In addition, the XRD pattern of V_2_O_5_ is shown in Supplementary Fig. S2.

### Structural and mechanical properties of the films

XRD was also employed to investigate the CMC/PEG blend structure and the roles of V_2_O_5_ and Cr_2_O_3_ in its structural characteristics. Figure 2 shows the patterns of virgin CMC/PEG, 0.5 $\text{wt\%}$ V_2_O_5_/blend, and 0.5–2.0 $\text{wt\%}$ Cr_2_O_3_/V_2_O_5_/blend. The pattern of the unfilled blend displays a broad peak in the region of $2\uptheta$ = 16–27° with maximum intensity at $2\uptheta$ = 20.9°. The appearance of this peak indicates the semicrystalline structure of the blend, i.e., the blend has crystalline regions inside an amorphous phase. Already, the XRD chart of the CMC polymer contains a wide peak around $2\uptheta$ = 22.13°^19^. This wide peak is certainly overlapped with the characteristic one of PEG (at $2\uptheta$ = 19.5°^16^). This semicrystallinity originates because the hydrogen bonds between PEG and CMC force the chains to form arranged lamellas throughout the amorphous parts^35^. A similar observation was noted for PVA/sodium alginate^36^. Introducing 0.5 $\text{wt\%}$ V_2_O_5_ and codoping with 0.5 $\text{wt\%}$ Cr_2_O_3_ decreases the intensity of the main peak. The fillers (V_2_O_5_ and Cr_2_O_3_) and the reactive groups of the matrix should have interacted and caused this change, where the amorphous areas inside the matrix became predominant. In other words, this note indicates that the filler inside the blend degrades the ordered (lamella) structures and increases the films' amorphousity^37^. It also indicates the good dispersion of these small amounts of the nanofillers inside the amorphous regions.

**Figure 3:**
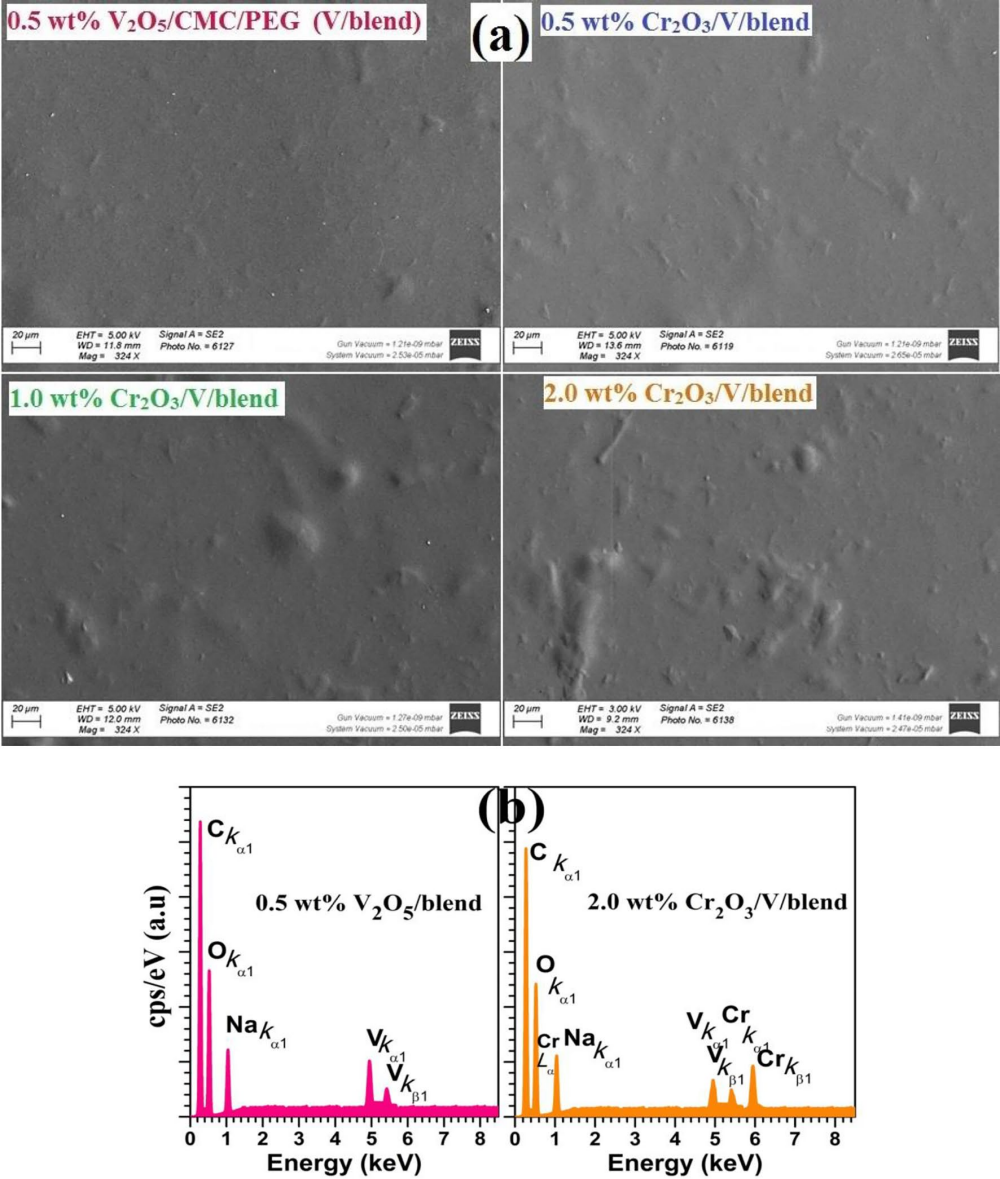
(**a**) FE-SEM images for 0.5 $\text{wt\%}$ V_2_O_5_/blend and 0.5–2.0 $\text{wt\%}$ Cr_2_O_3_/V/blend (scale bar: 20 µm), (**b**) EDAX spectra of 0.5 $\text{wt\%}$ V_2_O_5_/blend and 2.0 $\text{wt\%}$ Cr_2_O_3_/V/blend.

However, with increasing the Cr_2_O_3_ NPs content, all of the Cr_2_O_3_ peaks emerge instead of the one at $2\uptheta$ = 24.45°, which certainly overlapped with the wide peak of the blend. In addition, the main peak's intensity significantly increased and shifted to $2\uptheta$ ≈ 21.2°. The nanofillers' interesting surface energy, which encourages particle aggregation when concentration increases, as shown in Fig. 3, may be responsible for this. Similarly, Alhazime^15^ detected the main halo peak of CMC/PVP modified with ZnS/NiO at 21.59°. The improvement in blend crystallinity may also be due to the local structural arrangements made by complex formation within the matrix. Raising the Cr_2_O_3_ content to 2.0 $\text{wt\%}$ led to this outcome. These results indicate the possible control of blend structure by inserting specific amounts of V_2_O_5_/Cr_2_O_3_ NPs^11^.

Figure 3a shows the surface images for the films taken by FE-SEM. The films are homogenous. nonporous, and crack-free. The fillers are uniformly distributed on the blend surface and form clusters as their content increases beyond 0.5 wt% V_2_O_5_. Also, the agglomerations are clearly presented in the 2.0 wt% Cr_2_O_3_/V-loaded film. The elemental chemical analysis for the blend filled with 0.5 wt% V_2_O_5_ and 2.0 wt% Cr_2_O_3_/V is depicted in Fig. 3b. The C and O are the main components of the CMC and PEG. Their peaks appear at 0.27 and 0.53 keV, respectively, arising from *K*_α1_ emissions. The peak at ~ 1.05 keV is owing to *K*_α1_ line of Na that is found in CMC. The peaks at ~ 4.95 and 5.40 keV confirm the presence of V in the 0.5 wt% V_2_O_5_-loaded film. In addition, the Cr element displays three emissions at 0.55 keV (*L*_α_), 5.4 keV (*K*_α_), and 5.95 keV (*K*_β_), respectively. Although XRD wasn't able to confirm the existence of V due to the small amount of added V_2_O_5_ and the detection limit of XRD, EDX spectra confirmed their presence.

**Figure 4:**
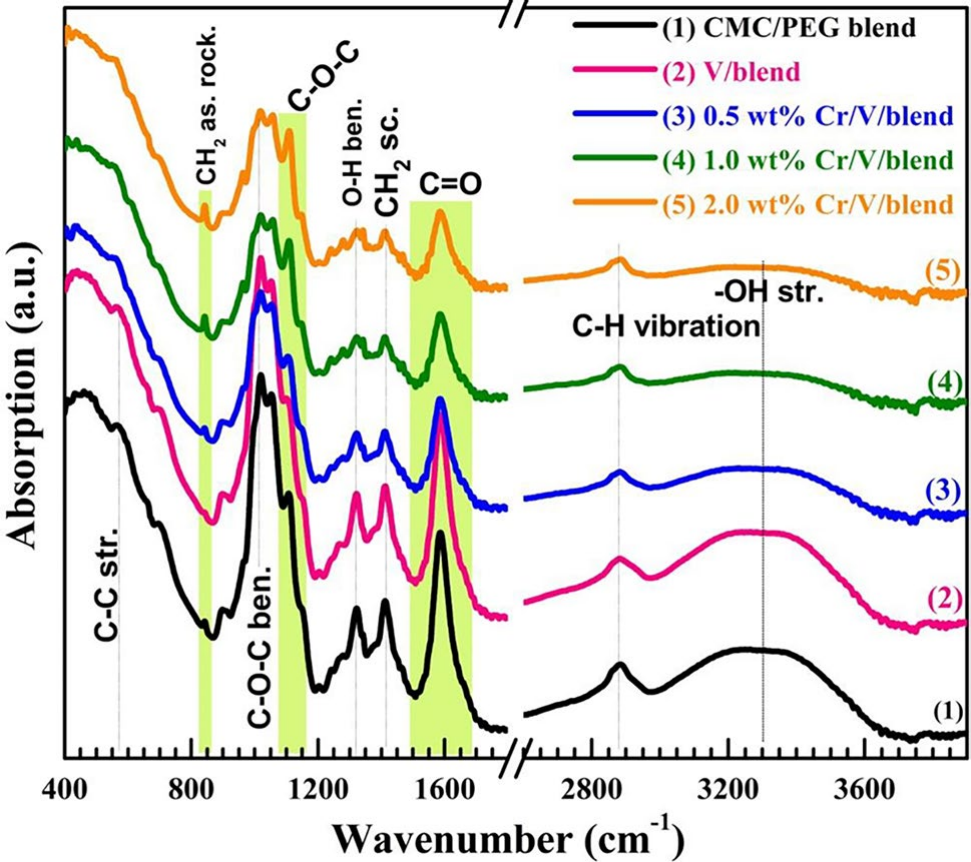
FTIR spectra of the blend, V_2_O_2_/blend, and Cr_2_O_3_/V_2_O_5_/blend nanocomposites.

Figure 4 displays the FTIR spectra of the blend and V_2_O_5_/Cr_2_O_3_/blend films. The main vibrational frequencies in these spectra are distinguished as follows: (i) A wide peak at about 3300 ${\text{cm}}^{-1}$ can be assigned to the O–H stretching in the blend. (ii) A minor peak at 2870 ${\text{cm}}^{-1}$ related to the vibration of H–C (aliphatic). (iii) The peaks located at 1022, 1317, 1421, and 1596 ${\text{cm}}^{-1}$ are owing to C–O–C and O–H bending, H_2_C scissoring, and vibrations of C=O of ${\text{COO}}^{-}$ group, respectively^11,20^. (iv) The vibrations of C–O stretching of ether groups are appearing at 1055 cm^–1^ and 1098 ${\text{cm}}^{-1}$^12^. (v) Finally, the tiny peaks at 841 and 580 ${\text{cm}}^{-1}$ are arising from the asymmetric rocking of CH_2_ and the stretching in C–C, respectively^6^. All of these functional groups are found in the CMC/PEG blend's structure. The spectra of the Cr_2_O_3_/V_2_O_5_/CMC/PEG films exhibit the same peaks but with reduced intensities compared to the blend. This suggests effective interactions between Cr_2_O_3_/V_2_O_5_ NPs and the blend molecules. The high tendency of OH to create charge-transfer complexes forces it to interact (physically) with V–O and Cr–O through chelating and the V_2_O_5_/Cr_2_O_3_ reinforcing effects^37^. Some minor peaks, however, at 841 and 1098 ${\text{cm}}^{-1}$ appear with decreasing intensity at 0.5 wt% V_2_O_5_ and 0.5 wt% Cr_2_O_3_/V_2_O_5_ content, then their intensity enhances with increasing Cr_2_O_3_ NPs ratio to 2.0 wt%. In addition, the intensity of 1098 ${\text{cm}}^{-1}$ band shifted to 1105 ${\text{cm}}^{-1}$ at 2.0 wt% Cr_2_O_3_/V_2_O_5_ filler content. A similar result was reported for PEO/CMC modified with MoO_3_ nanoblets^12^. No new bands are observed in FTIR spectra of V_2_O_5_ and V_2_O_5_/Cr_2_O_3_-doped films. This confirms the physical interactions between these nanofillers and blend molecules^11^.

**Figure 5:**
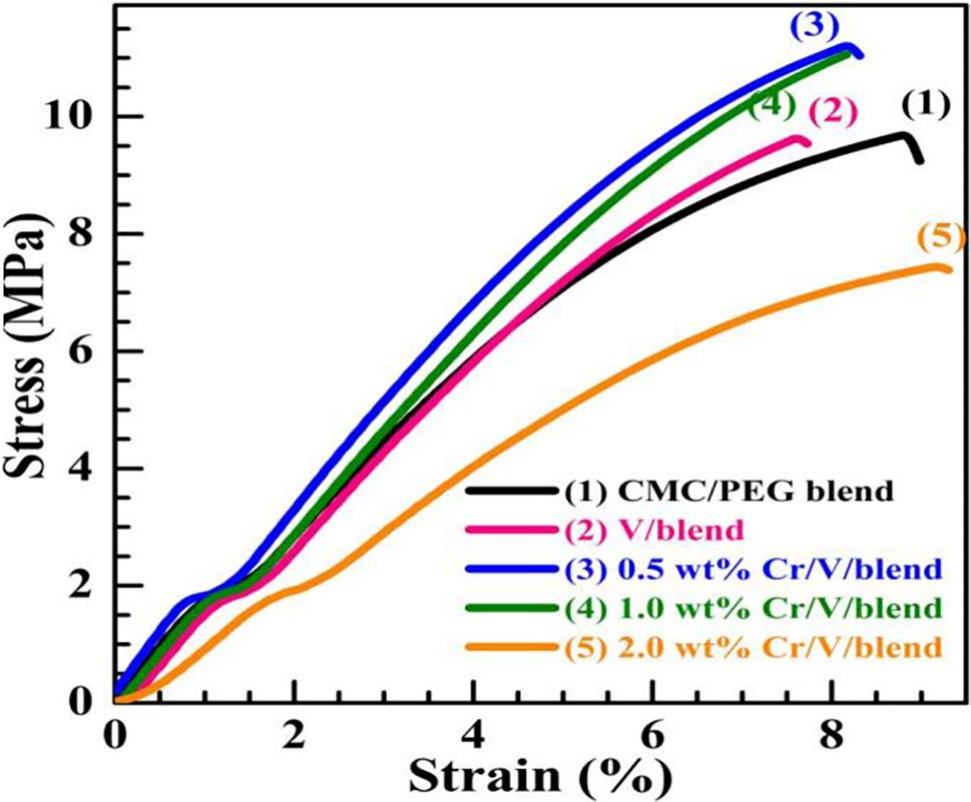
The tensile testing of the films.

The stress–strain curves of the films are shown in Fig. 5, and the values of the mechanical parameters (${\sigma }_{\text{M}}$, ${\varepsilon }_{\text{M}}$, ${\sigma }_{\text{B}}$, ${\varepsilon }_{\text{B}}$, and toughness) of the films are summarized in Table 1. The blend has ${\sigma }_{\text{M}}$ = 9.75 MPa which is consistent with the value reported for CMC (10.33 MPa)^8^. The values of ${\sigma }_{\text{M}}$, ${\varepsilon }_{\text{M}}$, ${\sigma }_{\text{B}}$ and ${\varepsilon }_{\text{B}}$ for CMC/PEG were improved slightly after filling with 0.5 wt% and 1.0 wt% Cr_2_O_3_/V_2_O_5_ NPs. Similarly, ${\sigma }_{\text{M}}$ of CMCS was improved after loading 10 wt% boehmite NPs^1^. The enhanced surface area of the added nanosized materials improves the interfacial area between the blend chains and these fillers. This simplifies transfer of the stress from the blend to the filler via the created interfaces and, hence, improves the ${\sigma }_{\text{M}}$. The decrease in ${\sigma }_{\text{M}}$ at 2.0 wt% Cr_2_O_3_/V_2_O_5_ arises from the agglomeration of fillers in the blend and the majority of the amorphous phase in the blend structure^38^. This agglomeration and the resulting decrease in ${\sigma }_{\text{M}}$ were the reasons for limiting the filler level to 2.0 $\text{wt\%}$. The ${\varepsilon }_{\text{B}}$ was reduced by doping with 0.5 $\text{wt\%}$ V_2_O_5_ then slightly increased with 1.0 $\text{wt\%}$ Cr_2_O_3_ NPs, but still smaller than that of the pure blend. The restriction of the mobility of the polymer chain after nanofiller insertion confirms their reinforcement effect. These reinforced, flexible, and lightweight films are needed for various everyday uses, such as electronic equipment.

**Table 1:** Mechanical parameters ($\text{mean }\pm \text{ standard error}$) of the films: ${\sigma }_{\text{M}}$ ($\text{tensile strength}$), ${\varepsilon }_{\text{M}}$ ($\text{strain at }{\sigma }_{\text{M}}$), ${\sigma }_{\text{B}}$ ($\text{stress at break}$), ${\varepsilon }_{\text{B}}$ ($\text{strain at break}$), and $\text{toughness}$.

$\text{Toughness}$ (MPa)	${\varepsilon }_{B}$ (%)	${\sigma }_{B}$ (MPa)	${\varepsilon }_{M}$ (%)	${\sigma }_{M}$ (MPa)	Film composition
130.6 ± 43	9.0 ± 2.0	9.2 ± 2.8	8.8 ± 1.9	9.75 ± 1.4	CMC/PEG (blend)
111.2 ± 14.0	7.8 ± 1.4	9.5 ± 5.3	7.6 ± 2.0	9.65 ± 1.8	0.5 wt% V_2_O_5_/blend (V/blend)
144.6 ± 10.4	8.35 ± 0.9	11.0 ± 4.7	8.2 ± 1.5	11.25 ± 2.6	0.5 wt Cr_2_O_3_/V/blend
132.3 ± 12.5	8.2 ± 0.9	11.0 ± 2.6	8.2 ± 1.4	11.0 ± 1.9	1.0 wt Cr_2_O_3_/V/blend
107.5 ± 9.5	9.3 ± 2.8	7.4 ± 2.6	9.2 ± 1.3	7.5 ± 1.4	2.0 wt Cr_2_O_3_/V/blend

### UV/vis–NIR spectra and optical features

**Figure 6:**
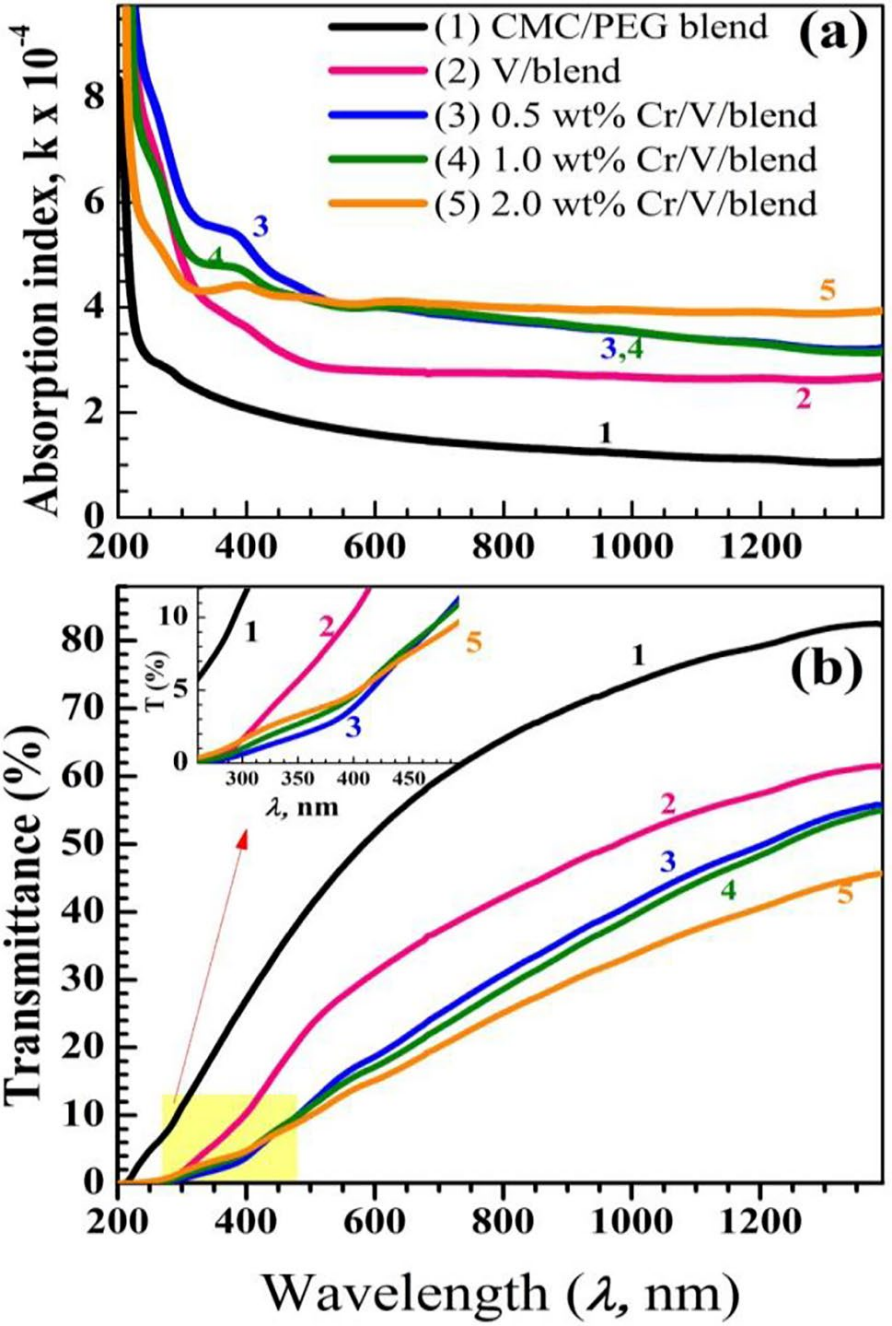
(**a**) absorption index spectra and (**b**) %transmittance of the films.

The optical absorption/transmittance spectra were recorded and studied to verify the applicability of the Cr_2_O_3_/V_2_O_5_/CMC/PEG films in optoelectronic applications. Figure 6a shows the absorption index (*k*) spectra that were determined from the recorded absorption ($\text{Abs}.$) and the incident wavelength ($\lambda$) as: $k= \frac{Abs. \lambda }{4d\pi }$^18^. The film thickness (*d*) is given in Table 2, and $\frac{Abs.}{d}=\alpha$ ($\text{the absorption coefficient}$). All films have small $k$ (*˂* 10^−3^) in the studied wavelength range. All nanocomposites exhibit higher *k* values relative to the blend. In the range $\lambda$ = 200–500 nm, the 0.5 $\text{wt\%}$ Cr_2_O_5_/V_2_O_5_/blend exhibits the highest value of *k*. At $\lambda$ *˃* 600 nm, the values of *k* increase with the fillers' content. New peaks are seen at ~ 400 and 620 nm in the $k$ spectra of CMC/PEG blends containing Cr_2_O_3_ NPs and they are attributed to the SPR or surface plasmon resonance of Cr_2_O_3_ NPs. Such phenomenon indicates the interesting optical features of the BPNC films and can be exploited in applications related to some optoelectronic systems and sensors^11^. The %transmittance values are depicted in Fig. 6b. The 0.5 $\text{wt\%}$ Cr_2_O_3_/V_2_O_5_/blend displays the lowest $\text{T\%}$ till $\lambda$* ≈* 420 nm, see the inset. At higher $\lambda ,\text{ T }(\text{\%})$ falls with raising the fillers content. For comparison purposes, $\text{T\%}$ values at 700 nm wavelength are inserted in Table 2. The added NPs act as scattering points which are able to reflect/absorb the incident radiation. The blend and nanocomposites show T in the range of 45–82%, which is suitable for many uses and applications.

**Table 2:** Film thickness ($d$), optical transmittance ($T\%$) at 700 nm, direct and indirect band gap (${E}_{g}^{d}$ and ${E}_{g}^{i}$), index of refraction ($n$), and band gap from ${\varepsilon }_{loss}^{op}$.

${E}_{g}^{loss}$ (eV)	$n$	${E}_{g}^{i}$ (eV)	${E}_{g}^{d}$ (eV)	$\text{T }(\text{\%})$	$d$ (mm)	Film composition
5.20	1.93	4.50	5.60	59.6	0.18	CMC/PEG (blend)
4.40	2.08	2.80	4.50	37.2	0.16	0.5 wt% V_2_O_5_/blend (V/blend)
4.10	2.17	2.50	4.00	24.9	0.15	0.5 wt Cr_2_O_3_/V/blend
4.70	2.05	3.05	4.70	22.8	0.19	1.0 wt Cr_2_O_3_/V/blend
4.90	1.97	3.80	5.20	20.1	0.16	2.0 wt Cr_2_O_3_/V/blend

**Figure 7:**
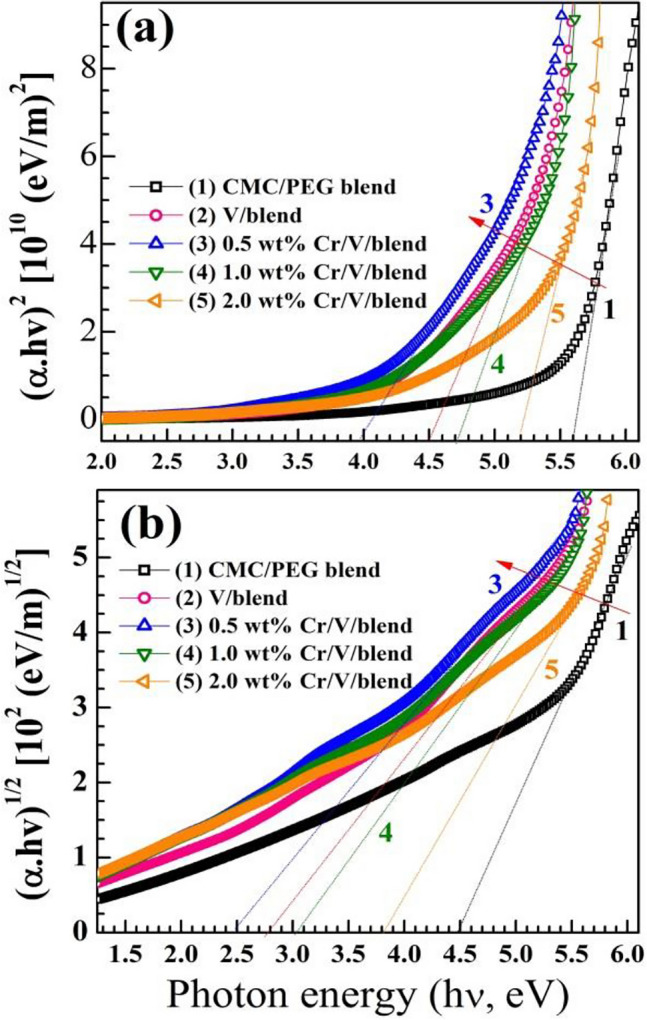
(**a**) ${E}_{\text{g}}^{\text{d}}$ and (**b**) ${E}_{\text{g}}^{\text{i}}$ of the films.

The possible optical transition and band gap (*E*_g_) of the films can be investigated considering Tauc and Davis–Mott relations: $(\alpha h\upsilon {)}^{2}=A(h\upsilon -{E}_{\text{g}}^{\text{d}})$ and $(\alpha h\upsilon {)}^{1/2}=B(h\upsilon -{E}_{\text{g}}^{\text{i}})$ for the allowed transitions (direct and/or indirect), where $h\upsilon$ is incident energy, A and B are constants, and ${E}_{\text{g}}^{\text{d}}$ and ${E}_{\text{g}}^{\text{i}}$ are the direct and indirect *E*_g_. The plots of $(\alpha h\upsilon {)}^{2}$ vs. $h\upsilon$ and $(\alpha h\upsilon {)}^\frac{1}{2}$ vs. $h\upsilon$ are illustrated in Fig. 7a,b. The nanocomposite films’ ${E}_{g}^{d}$ (${E}_{g}^{i}$) values decreased from 5.6 (4.5) eV to 4.0 (2.5) eV upon loading 0.5 wt% Cr_2_O_3_/0.5 $\text{wt\%}$ V_2_O_5_. Similarly, V_2_O_5_ NPs created inside the chitosan/PVA by laser ablation reduced the ${E}_{\text{g}}^{\text{d}}$ of the blend from 5.72 to 3.72 eV^27^. Incorporating MoO_3_ nanofillers (2.0–8.0 $\text{wt\%}$) has reduced ${E}_{g}^{d}$ (${E}_{g}^{i}$) of CMC (80%)/PEO(20%) blend from 5.1 (3.2) eV to 3.24 (1.65) eV^12^. The observed decrement in ${E}_{g}^{d}$ (${E}_{g}^{i}$) is assigned to the strong interaction of the host matrix with the fillers which produce defects and localized states inside the CMC/PEG gap and change the disorder degree of the host blend.

In addition, this decrement may result because the fillers can form a connected network in the structure of the blend and simplify the charge-carriers motion^9,12^. In the literature, it was reported that adding of 10 $\text{wt\%}$ Zn_0.9_Cu_0.1_S to the blend composed of PVA (0.7)/CMC (0.15)/PEG (0.15) shrink its ${E}_{\text{g}}^{\text{d}}$ (${E}_{\text{g}}^{\text{i}}$) from 5.87 (5.31) eV to 4.47 (3.65) eV^9^. In addition, loading 6.0 $\text{wt\%}$ CdO NPs inside PVA/CMC matrix has reduced its ${E}_{\text{g}}^{\text{d}}$ (${E}_{\text{g}}^{\text{i}}$) from 5.65 (4.57) eV to 5.07 (3.91) eV^24^. However, at 2.0 $\text{wt\%}$ Cr_2_O_3_/V_2_O_5_, a widening of ${E}_{\text{g}}^{\text{d}}$ (${E}_{\text{g}}^{\text{i}}$) to 5.2 (3.8) eV is observed. This blue shift may have happened due to NPs agglomeration and improving the ordering and crystallinity inside the blend, as seen from the XRD patterns of the samples. Similarly, doping with 0.5–1.5 $\text{wt\%}$ TiO_2_ NPs has reduced the ${E}_{\text{g}}^{\text{d}}$ (${E}_{\text{g}}^{\text{i}}$) of the PVA/PVP/CMC blend from 5.43 (4.97) eV to 5.27 (4.72) eV, then they increased to 5.34 (4.77) eV at 3.0 wt% TiO_2_ loading^23^. In contrast, loading 0.1 $\text{wt\%}$ SrTiO_3_/carbon nanotubes widened the ${E}_{\text{g}}^{\text{d}}$ (${E}_{\text{g}}^{\text{i}}$) of the polyvinylidene from 5.56 (5.27) eV to 5.70 (5.50) eV. Further increase in the filler level to 0.7 wt% decreased ${E}_{\text{g}}^{\text{d}}$ (${E}_{\text{g}}^{\text{i}}$) of the polymer to 5.53 (5.3) eV^5^. These observations make it possible to tune or engineer the ${E}_{\text{g}}^{\text{d}}$ and ${E}_{\text{g}}^{\text{i}}$ for various optoelectronic and electrochemical applications such as photocatalysis, solar cells, and sensors.

**Figure 8:**
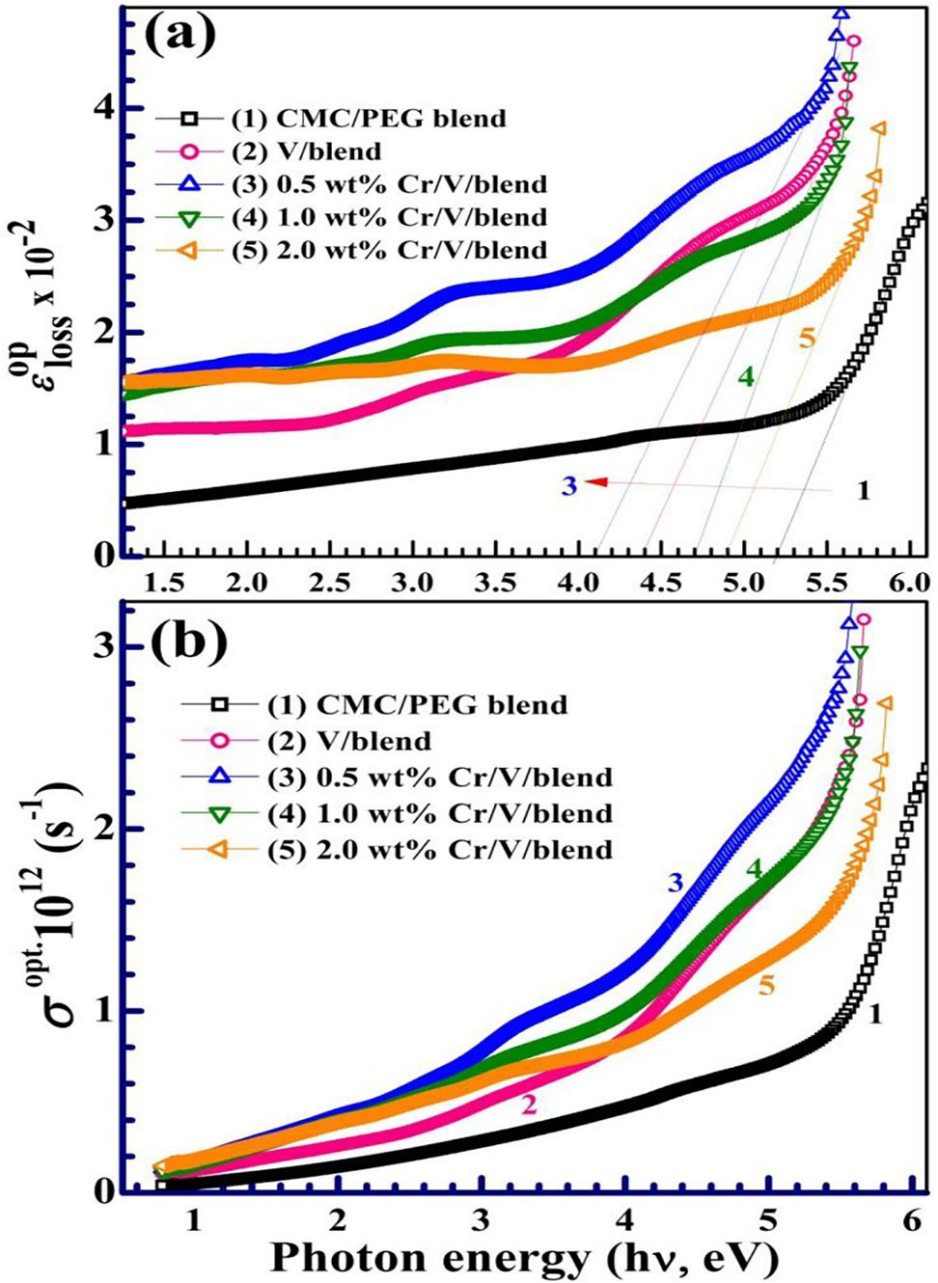
**a**,**b** Optical dielectric loss (${\varepsilon }_{loss}^{op}$) and the optical conductivity of the samples.

The index of refraction (*n*) is an essential parameter for design of the optical device. The *n* values were derived from the ${E}_{\text{g}}^{\text{d}}$ using the relation: $\frac{{n}^{2}-1}{{n}^{2}+2}=1-\sqrt{\frac{{E}_{\text{g}}^{\text{d}}}{20}}$^39^, see Table 2. As can be noted, the behavior of *n* with doping is the reverse to that of *E*_g_ values. The *n* value increased from 1.93 to 2.17 and this means that the films' reflectivity is improved, where the fillers act as scattering points. A similar finding was reported by Menazea et al.^27^ where the laser-induced V_2_O_5_ increased *n* of chitosan/PVA from 2.13 to 3.32. NPs The dielectric loss ${\varepsilon }_{loss}^{op}$ can be derived from *n* values and *k* spectra as ${\varepsilon }_{loss}^{op}$ = $2nk$^40^. Figure 8a shows the behavior of ${\varepsilon }_{loss}^{op}$ with $h\upsilon$, as an alternative model to Tauc and Davis–Mott relation for estimating the *E*_*g*_ value^41^. ${\varepsilon }_{loss}^{op}$ shows the role of the photon energy in the transition of the electrons. Extending the linear portions in the ${\varepsilon }_{loss}^{op}$ plots the $h\upsilon$ axis can be used to obtain the ${E}_{g}^{loss}$ values, as listed in Table 2. The ${E}_{g}^{loss}$ also decreases from 5.2 to 4.1 eV at 0.5 $\text{wt\%}$ Cr_2_O_3_/0.5 $\text{wt\%}$ V_2_O_5_ content, then increases to 4.9 eV at 2.0 $\text{wt\%}$ Cr_2_O_3_/0.5 $\text{wt\%}$ V_2_O_5_, which is consistent with the change in ${E}_{g}^{d}$. Therefore, the ${E}_{g}^{loss}$ and Tauc and Davis–Mott model can be used effectively to evaluate the *E*_*g*_. A similar consistency between the two approaches was reported for PVA/PEG solid polymer electrolyte films^40^ and AlPO_4_/PVA/PEO blend^42^.

The optical conductivity (${\sigma }_{op.}$) of the can be determined from the relation^43^: ${\sigma }_{op.}= \frac{\alpha nC}{4\pi }$, where *c* is the speed of light. Figure 8b shows the curves of ${\sigma }_{op.}$ vs. $h\upsilon$. All filled films exhibit enhanced ${\sigma }_{op.}$ in comparison with the unfilled blend. Moreover, the ${\sigma }_{op.}$ values enhanced with filler loading till 0.5 $\text{wt\%}$ Cr_2_O_3_/V_2_O_5_. It increases linearly with *hυ* till ~ 3.0 eV, and then the increase is slowdown. At *hυ* ˃ 5.0 eV, the UV photons cause a sharp increase in ${\sigma }_{op.}$ values by exciting the charge carriers (electrons) to take part in the conduction process^44^.

### Electrical and dielectric features of nanocomposite films

#### AC conductivity

**Figure 9:**
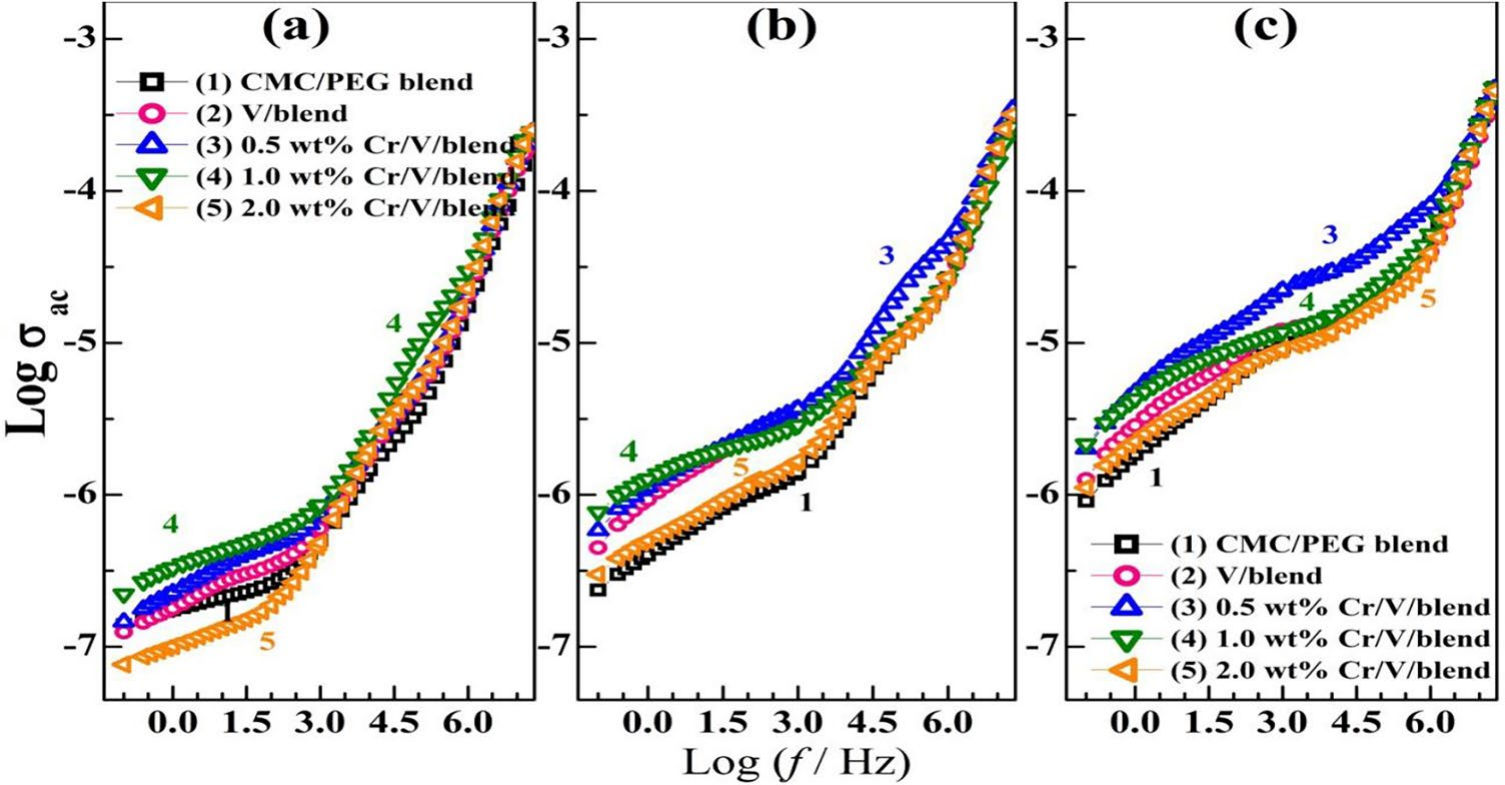
(**a**–**c**) Dependence of the ac conductivity on *f* for CMC/PEG, V_2_O_5_/blend, and Cr_2_O_3_/V_2_O_5_/blend at temperatures of (30, 50, 70 °C).

The BPNC' conductivity depends on the polymer's polarity, chemical nature of the fillers, doping ratio, particle size, dispersion, and the interfacial interactions between the chains of the polymer with the loaded particles^1^. Figure 9a–c shows the variation of ${\sigma }_{ac}$ with the applied *f*, Log (${\sigma }_{ac}$) vs. Log (*f*), at different temperatures (30, 50 and 70 °C). The dispersion of ${\sigma }_{ac}$ at low *f* is assigned to the interiorizations or spatial charges^11^. The ${\sigma }_{ac}$ of the CMC/PEG films is improved after loading the NPs till specific concentration of Cr_2_O_3_, (1.0 wt% Cr_2_O_3_ at 30 °C, and 0.5 $\text{wt\%}$ Cr_2_O_3_ at 50 and 70 °C) where the Cr_2_O_3_ molecules can connect the gaps among the localized states and facilitate or simplify the charge carrier mobility. The increment of the amorphous portion throughout the blend after NPs incorporation as seen from the XRD results, the uniform distribution of the fillers, and the interfacial polarization among the chains of the blend and V_2_O_5_/Cr_2_O_3_ improve the films' conductivity. At 2.0 $\text{wt\%}$ Cr_2_O_3_/V_2_O_5_, the agglomeration of the NPs reduces the interaction zone with the blend, and alters the charge carrier and defect numbers at the film surface due to the interactions among the Cr_2_O_3_/V_2_O_5_ NPs inside the matrix^45^. Consequently, a substantial decrement of the interfacial polarization. Similar observations were noted in the system of carboxymethyl chitosan/cashew gum filled with boehmite NPs^1^, and CMC/PEO loaded with TiO_2_/MWCNT^46^.

The ${\sigma }_{ac}$ is almost *f*-independent at lower *f* regions and increases rapidly above 10^3^ Hz which means that ${\sigma }_{ac}$ obeys Jonscher’s power relation: ${\sigma }_{(\omega )}= {\sigma }_{dc}+A{\omega }^{s}$, where ${\sigma }_{dc}$, *A* and *s* are the conductivity at $f = 0$, a constant, and frequency exponent factor, respectively, and $\upomega$ = 2п*f*. This can be assigned to the hopping of charge carriers that acquire sufficient energy with increasing *f* and can hop through the conducting clusters and overcome the resistive grain boundaries^47^. In addition, the ${\sigma }_{ac}$ values at a certain value of *f* increase with heating from 30 to 70 °C, where the charge carrier mobility affected by the provided thermal energy. Also, the transit sites number increases with heating. Hence, the excited charge carriers can move through the matrix and overcome the energy barrier and take part in the conduction process^48^. Similarly, at 10 kHz, the ${\sigma }_{ac}$ of CMCS nanocomposites increased from about 0.4 × 10^−5^ at 30 °C to 0.34 × 10^–4^ S/cm at 90 °C^1^. The enhancement in the blend conductivity after filling with V_2_O_5_/Cr_2_O_3_, and with increasing *f* and temperature make the nanocomposite films more relevant for potential applications, including the optoelectronic devices and solid-state batteries.

#### Dielectric constant, loss, and energy density

**Figure 10:**
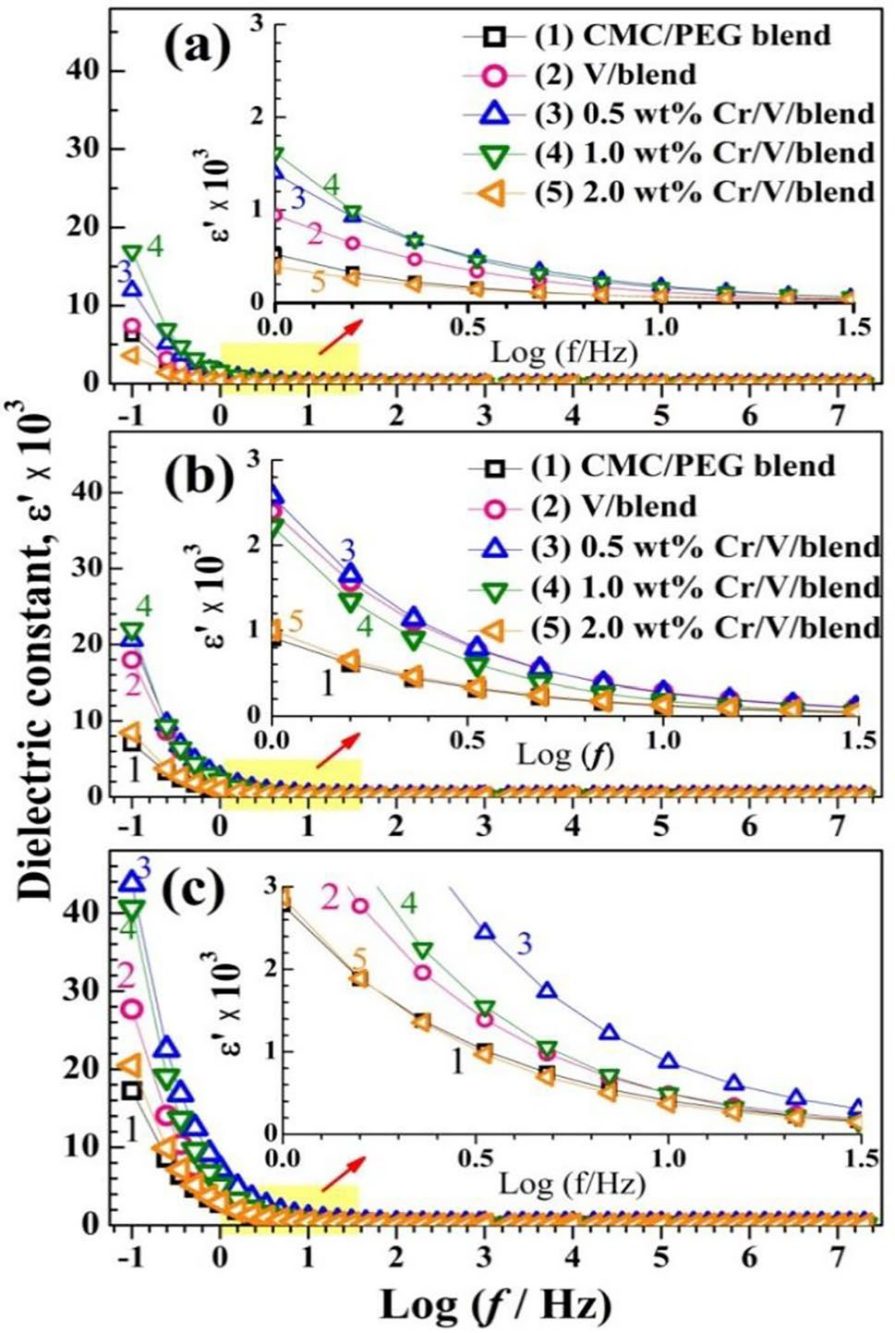
(**a**–**c**) Variation of ${\varepsilon }{\prime}$ with $f$ at temperatures of (30, 50, 70 °C).

**Figure 11:**
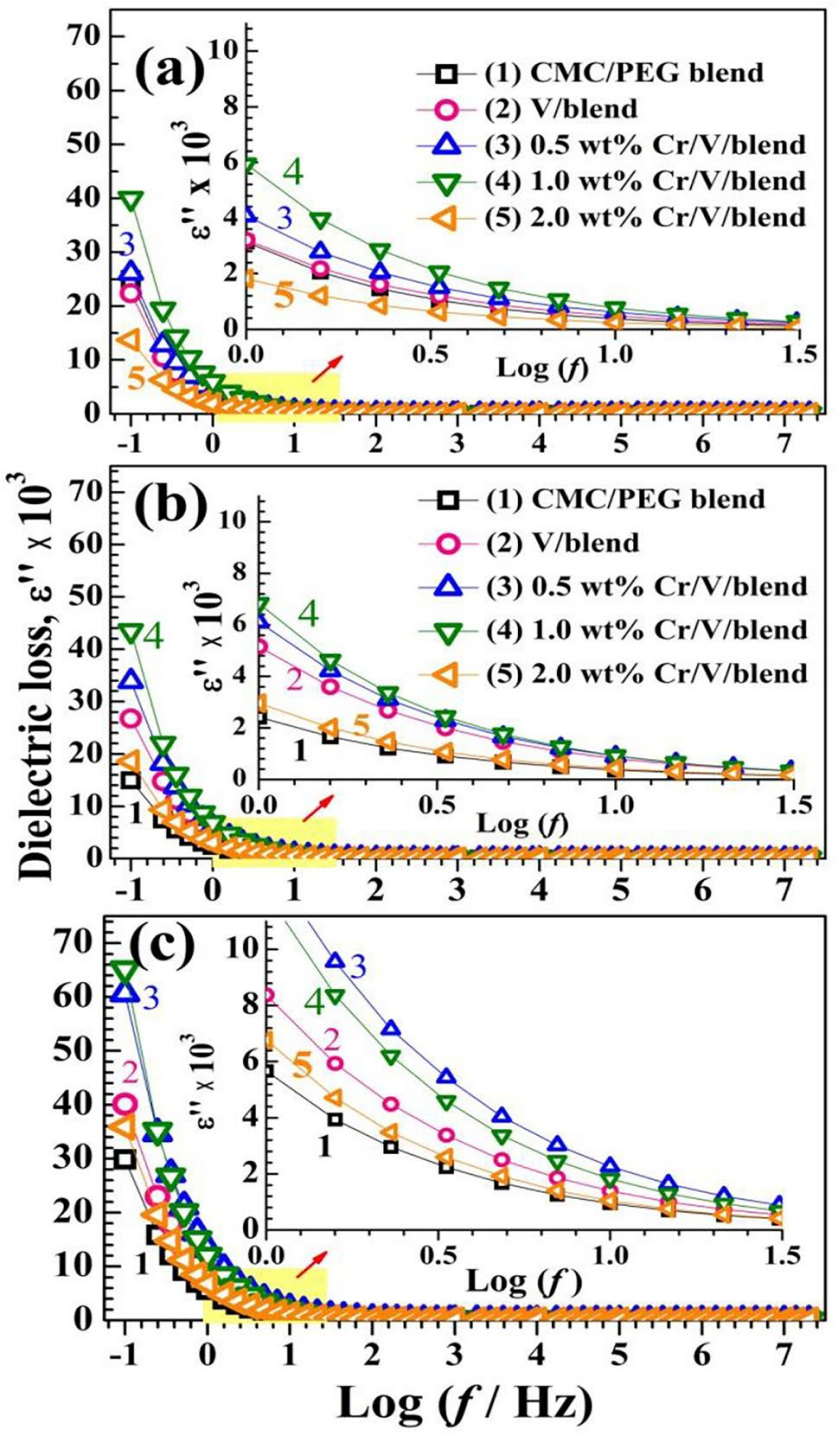
(**a**–**c**) Variation of ${\varepsilon }^{{\prime}{\prime}}$ with $f$ at 30, 50, and 70 °C.

The charge storage capacity can be determined through the dielectric permittivity analysis. The dielectric permittivity: ${\varepsilon }^{*}= {\varepsilon }{\prime}- j{\varepsilon }^{{\prime}{\prime}}$, where ${\varepsilon }{\prime}$ and ${\varepsilon }^{{\prime}{\prime}}$ represent the dielectric constant (the ability of the materials to store the energy due to all possible polarizations) and the dielectric loss (the energy dissipated in each cycle of applied *f*), respectively. The interfacial and dipolar polarizations are present in the unfilled matrix because it comprises a lot of polar groups and its constituents are of different conductivities. Figures 10 and 11 display the spectra of ${\varepsilon }{\prime}$ and ${\varepsilon }^{{\prime}{\prime}}$ for CMC/PEG, V_2_O_5_/blend and Cr_2_O_3_/V_2_O_5_/blend at three different temperatures; 30, 50, and 70 °C. Both ${\varepsilon }{\prime}$ and ${\varepsilon }^{{\prime}{\prime}}$ display the same behavior with *f*; a fast decrement with *f* and are small or steady in the higher frequency region (*f*-independent).

These figures illustrate that a non-Debye type of behavior, where the space-charge accumulation is the reason behind the higher value of ${\varepsilon }{\prime}$ at the lower *f* side without any relaxation peaks with increasing *f*. The large ${\varepsilon }^{{\prime}{\prime}}$ at low *f* is owing to the interfacial polarization (also known as Maxwell–Wanger–Sillers effect), where numerous charge carriers/dipoles have the time to be oriented in the alternative field direction, exhausting a significant energy value^16^.

The decrement of ${\varepsilon }{\prime}$ and ${\varepsilon }^{{\prime}{\prime}}$ values as *f* increases is because the charge carrier and the chain segments can't follow the field due to the limited time. Therefore, the contribution of charge carriers to ${\varepsilon }{\prime}$ with increasing *f* is diminished. Both ${\varepsilon }{\prime}$ and ${\varepsilon }^{{\prime}{\prime}}$ are sensitive to the temperature and fillers' concentration. At 30 °C, their values increased with doping till 1.0 $\text{wt\%}$ Cr_2_O_3_ NPs. This could be attributed to that the interactions between V_2_O_5_, Cr_2_O_3_ and CMC/PEG chains significantly affects the parallel ordering of the C–O–C, –OH, and C=O groups^12^. Seemingly, it could be possible to fabricate electrochemical devices of high performance using these nanocomposite films. The observed decrease in *ε*′ at filler concentration of 2.0 $\text{wt\%}$ Cr_2_O_3_/V_2_O_5_ may be explained in terms of the electrostatic interactions inside this heterogeneous structure which may result in a decrease in the interfacial polarization by creating microcapacitors and hence ε′ reduced^49^. Similar results were reported for Zn_0.95_V_0.05_S/CMC/PVP/PEG^16^ and ZnO/TiO_2_/PEO/CMC nanocomposites^50^. The decrease in ${\varepsilon }^{{\prime}{\prime}}$ values with increasing filler content beyond 0.5 wt% V_2_O_5_ and 0.5 $\text{wt\%}$ Cr_2_O_3_ is related to the decrement in *σ*_ac_ which is directly related to ${\varepsilon }^{{\prime}{\prime}}$. Raising the temperature from 30 to 70 °C increases the supplied thermal energy to the blend system which can dissociate any coupled charges. In addition, the decrease of the polymer viscosity with heating facilitates the dipole/charge carrier motion to orient them in the field direction. This leads to intensifying the polarization and increasing the ${\varepsilon }{\prime}$^51^.

**Figure 12:**
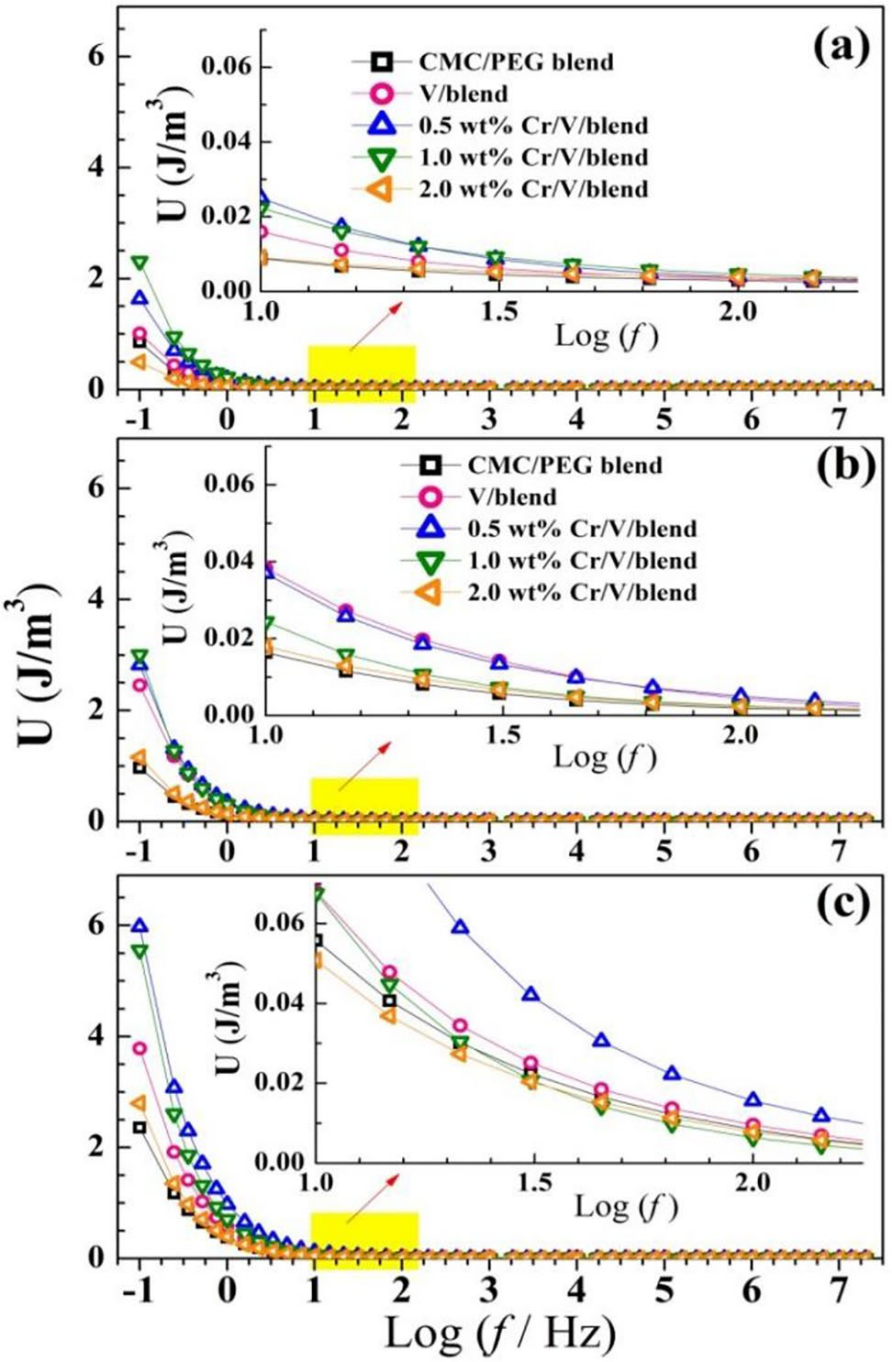
(**a**–**c**) Energy density of CMC/PEG, V_2_O_5_/blend, Cr_2_O_3_/V_2_O_5_/blend at temperatures of (30, 50, 70 °C).

The stored energy in a specific volume (energy density, *U*) is related ${\varepsilon }{\prime}$, free-space permittivity (${\varepsilon }_{o}$), and the electric filed (*E*) according the relation: $U= \frac{1}{2}{\varepsilon }_{o}{\varepsilon }{\prime}{E}^{2}$^22^. Figure 12 shows *U* vs. Log (*f*) for CMC/PEG loaded with V_2_O_5_ and Cr_2_O_3_/V_2_O_5_ at different temperatures. U (*f*) of the blend increased upon doping with 0.5 and 1.0 wt Cr_2_O_3_/V_2_O_5_. The insets of Fig. 12 show the exponential decrease of *U* with *f*, but the *U* values significantly improved with heating. At very low *f*, *U* at 50 °C in the range of 1–3 J/m^3^ and at 70 °C is in the range of 2–6 J/m^3^. The observed improvements in the U values make the doped blend best suitable for energy-related uses^3^.

#### Dielectric moduli and Argend plots

**Figure 13:**
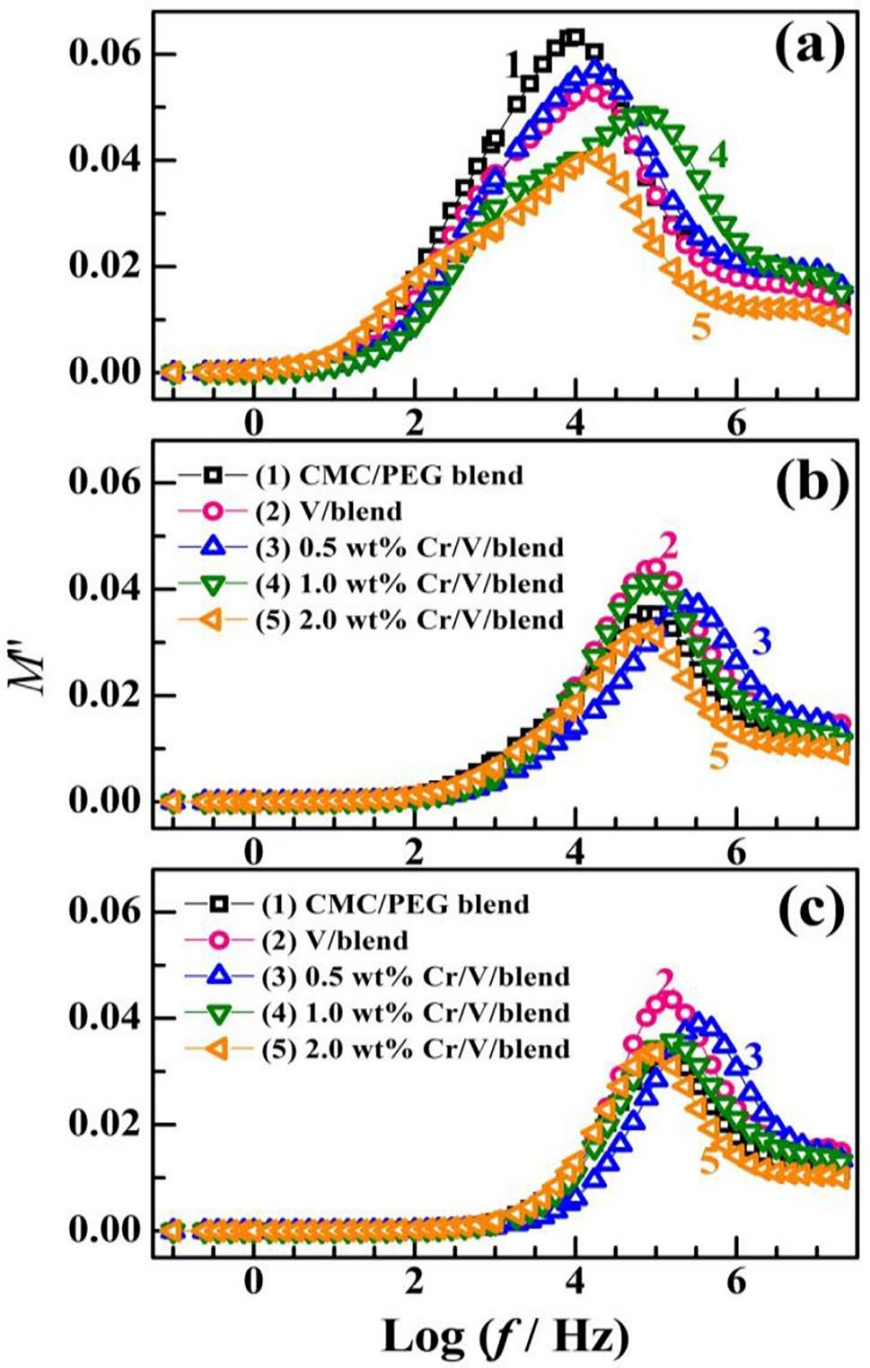
(**a**–**c**) Dielectric modulus ${M}{\prime}{\prime}$ distribution of CMC/PEG blend and blend loaded with V_2_O_5_, and Cr_2_O_3_/V_2_O_5_ NP at temperatures of (30, 50, 70 °C).

Supplementary Figure S3 and Fig. 13 display the dependence of the actual (${M}{\prime}$) and the (${M}^{{\prime}{\prime}}$) fictitious modulus on *f* for the unfilled blend and Cr_2_O_3_/V_2_O_5_/blend films at different temperatures. Both the two moduli display virtually very low value at lowest *f* values, which indicates that the charge carriers are constrained in the blend and that the effect of electrode polarization is minimized. ${M}{\prime}$ displays a reverse behavior for ${\varepsilon }{\prime}$, so ${M}{\prime}$ is the retro quantity of ${\varepsilon }{\prime}$^23^. ${M}{\prime}$ grow linearly with *f* in the middle region of the applied frequencies and is nearly constant at higher *f*. This linear increase is owing to the charge carriers possess motilities of short range and the resulted conductivity^23^. At 30 °C, ${M}{\prime}$ decreases with increasing the fillers' content, this affirms the role of V_2_O_5_/Cr_2_O_3_ in the film's conductivity. Increasing the temperatures to 50 and 70 °C results in a decrement in the ${M}{\prime}$ values. The observed relaxation peak in the *M″* spectrum (Fig. 13) indicates a decent ionic conductivity contribution in the films^12^. At 30 °C, the relaxation peaks in the ${M}^{{\prime}{\prime}}$ spectra shifted to a relatively higher *f* after doping with Cr_2_O_3_/V_2_O_5_ nanofillers. Also, the maximum peak lowered considerably with increasing the fillers content.

On the low *f* side of the relaxation peak, the ions can move over long distances by jumping among the neighbor sites. On the high *f* side of the peak, the charge carriers or ions can only move inside their wells. In other words, a phase transition occurred due to the change in dipole mobility from long to short ranges^31^. The dielectric relaxation time (τ ≈ 1/(2π*f*_max_) decreased from 1.6 × 10^−5^ s for the blend to 0.5 × 10^–5^ s for 2.0 wt% Cr_2_O_3_/V_2_O_5_/blend film. At elevated temperatures, the relaxation peak position and intensity follow a non-monotonic trend with the fillers content.

**Figure 14:**
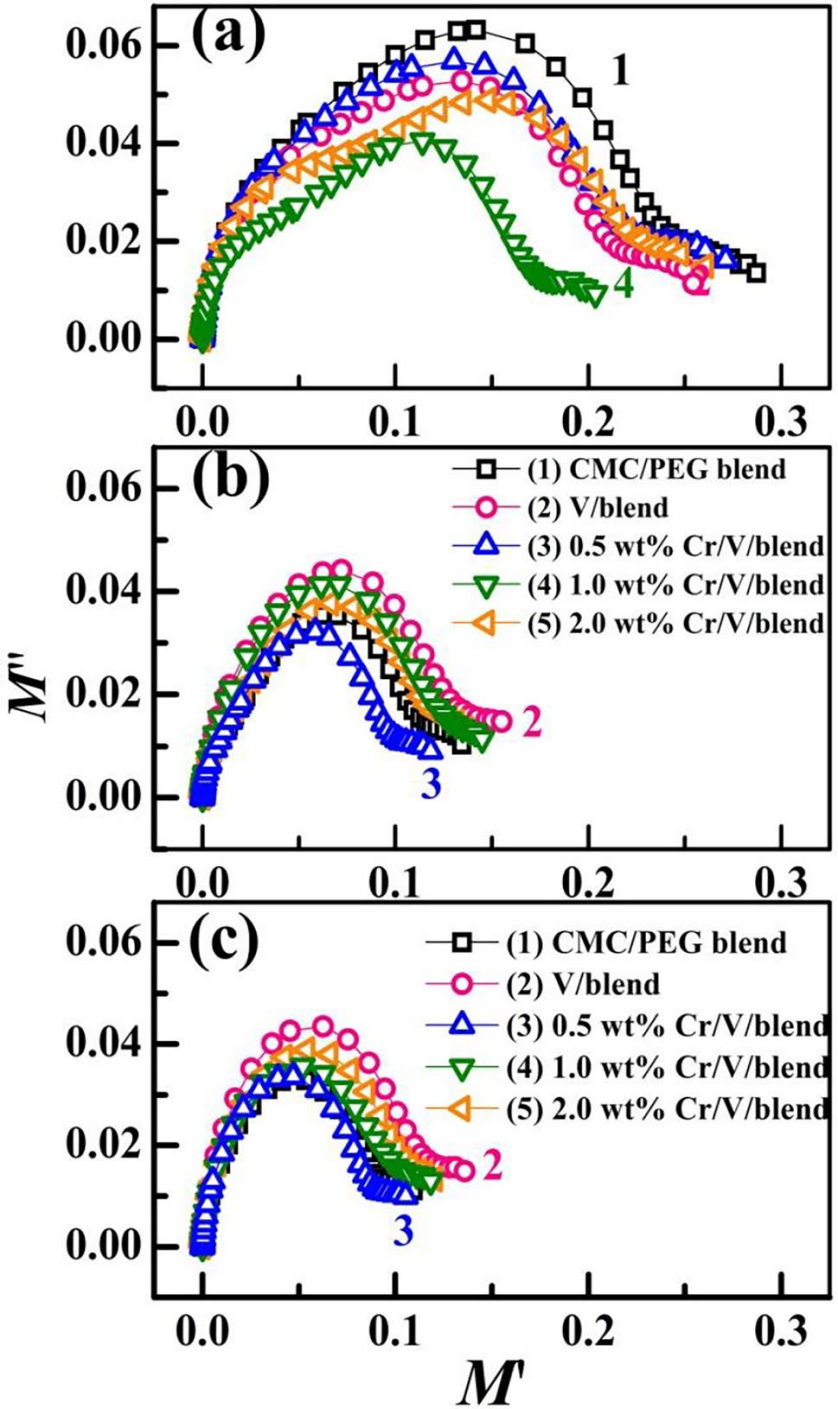
(**a**–**c**) ${M}{\prime}$ vs. ${M}^{{\prime}{\prime}}$ curves of CMC/PEG blend and blend loaded with V_2_O_5_, and Cr_2_O_3_/V_2_O_5_ NP at temperatures of (30, 50, 70 °C).

The variation of ${M}{\prime}$ vs. ${M}^{{\prime}{\prime}}$ is shown in Fig. 14, of CMC/PEG blend and nanocomposites display semicircles with centre positions not on the horizontal ${M}^{{^{\prime}}}$ axis. This trend and the observed decrease in $\tau$ indicate the non-Debye type of the observed relaxation, which can be owing to the existence of relaxation mechanisms, various types of polarization, and complex interactions among the dipoles and ions^23,24^. At 30 °C, the semicircle diameters reduced with increasing the fillers content. Also, they increased significantly with heating the samples from 30 to 70 °C. The sample with the smaller diameter possesses the lowest resistance to the mobile charges^52,53^. The observed single semicircles inside the blend and Cr_2_O_3_/V_2_O_5_/blend nanocomposites denote singular relaxation^6^

## Conclusion

Cr_2_O_3_/V_2_O_5_/CMC/PEG BPNC was successfully prepared by chemical solution techniques. TEM/SEM analyses confirmed the nanostructuration of the fillers. XRD, FE-SEM and EDX analyses confirmed the successful doping with 0.5 $\text{wt\%}$ V_2_O_5_ and codoping with 0.5–2.0 $\text{wt\%}$ Cr_2_O_3_ NPs into the blend. The fillers tend to agglomerate when Cr_2_O_3_ NPs is 1.0 wt% or more. FTIR spectroscopy revealed the complexation and the physical interactions of the fillers with the reactive groups (C–O–C, OH, C=O) of the blend. This interaction and the uniform filler distribution improved the tensile strength from 9.75 to 11.25 MPa and the stress at break from 9.2 to 11 MPa. The Cr_2_O_3_ content of 0.5 wt% and 2.0 wt% showed a higher absorption index in the UV and IR regions, respectively. All films showed transmittance in the range of 45–82%. ${E}_{g}^{d}$ (${E}_{g}^{i}$) decreased from 5.6 (4.5) eV to 4.0 (2.5) eV at 0.5 $\text{wt\%}$ Cr_2_O_3_, then increased to 5.2 (3.8) eV at 2.0 $\text{wt\%}$ Cr_2_O_3_ content. The ${E}_{g}$ derived from ${\varepsilon }_{loss}^{op}$ exhibited similar behavior as ${E}_{g}^{d}$. In addition, the index of refraction improved from 1.93 to 2.17. Both the optical and AC conductivities depended on the filler type and content. The dielectric constant, dielectric loss, and energy density were enhanced with Cr_2_O_3_ ratio of 1.0 $\text{wt\%}$ and also significantly improved with raising the temperature from 30 to 70 °C. The ${M}^{{\prime}{\prime}}$ spectra showed a relaxation peak of a non-Debye type. The Cole–Cole plots of CMC/PEG blend and nanocomposites displayed semicircles of reduced diameter as the temperature and filler content increased. The possible engineer or control of the optical features, mechanical properties, and the electrical properties by a certain amount of V_2_O_5_ and Cr_2_O_3_ makes these nanocomposites the best candidates for optoelectronics, sensors, and energy-related applications.

### Supplementary Information

Supplementary Information.

## Data Availability

The authors declare that all data generated or analyzed during this study are included in this published article and its supplementary materials file.
