# Supplementary material for: Tuning the band gap, optical, mechanical, and electrical features of a bio-blend by Cr2O3/V2O5 nanofillers for optoelectronics and energy applications
Source: Sci Rep. 2024 May 31;14:12537. doi: 10.1038/s41598-024-62643-6 (PMC11143206; doi:10.1038/s41598-024-62643-6)
Supplement: Supplementary file 1 — Supplementary Information. [file 41598_2024_62643_MOESM1_ESM.pdf]

Tuning the band gap, optical, mechanical, and electrical features of a bio-blend by Cr_2O_3/V_2O_5 nanofillers for optoelectronics and energy applications

Tarek I. Alanazi[1], Raghad A. Alenazi[1], Adel M. El Sayed[2*]

[1]Department of Physics, College of Science, Northern Border University, Arar 73222, Saudi Arabia

[2]Physics Department, Faculty of Science, Fayoum University, El-Fayoum 63514, Egypt

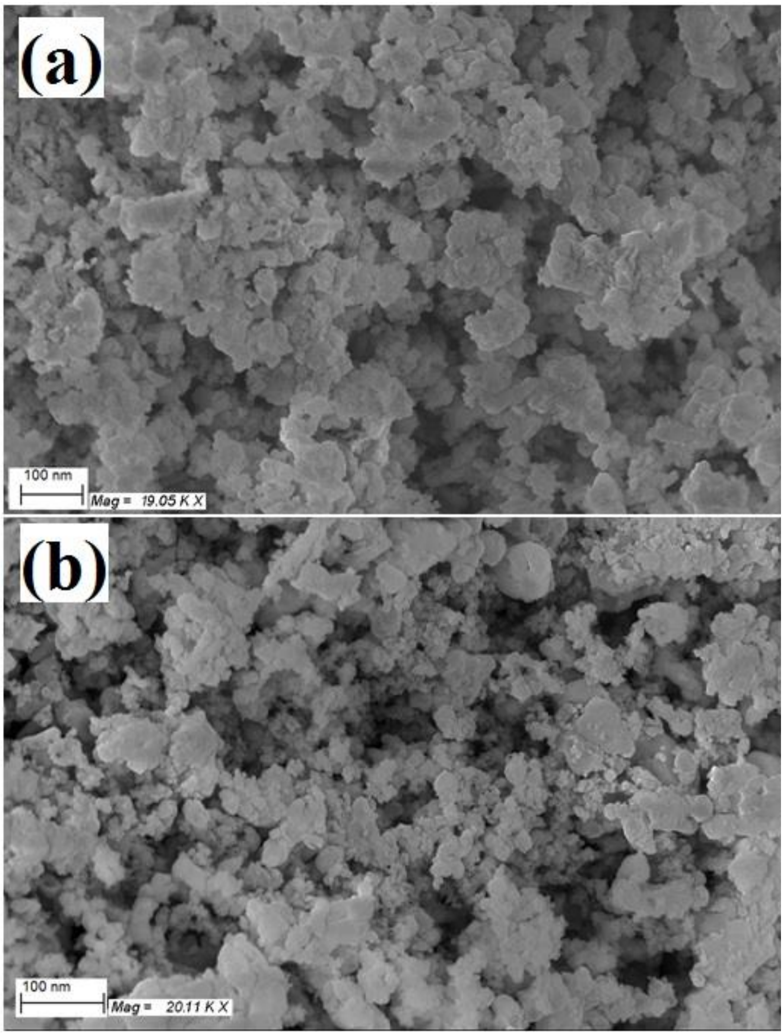

Fig. S1: FE-SEM images for (a) V_2O_5, (b) Cr_2O_3 NPs.

Phase name	Chemical formula	FOM	Phase reg. detail	Space Group	DB Card Number
Shcherbianite	V2 O5	1.239	S/M:COD	31 : Pmn21	1011291

Phase Data View

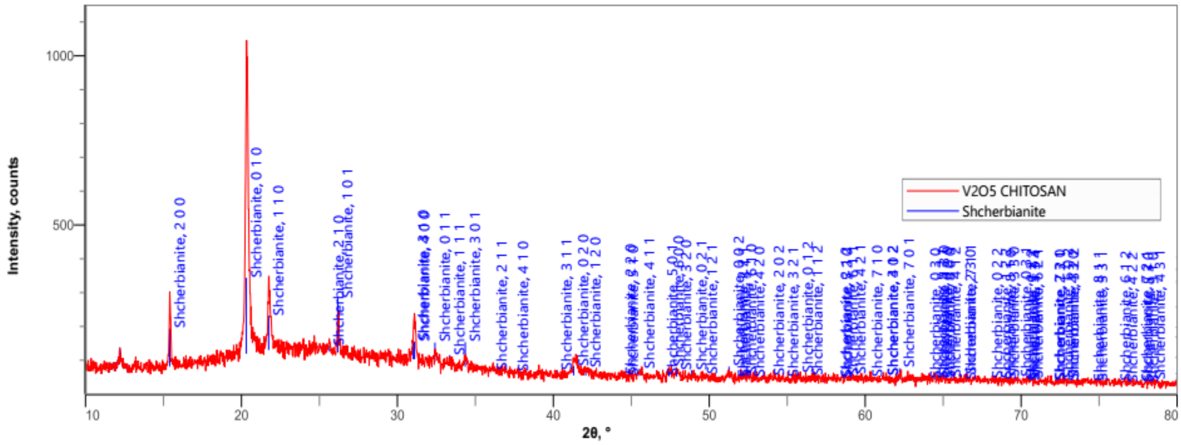

Fig. S2: XRD of the as-synthesized V_2O_5 NPs.

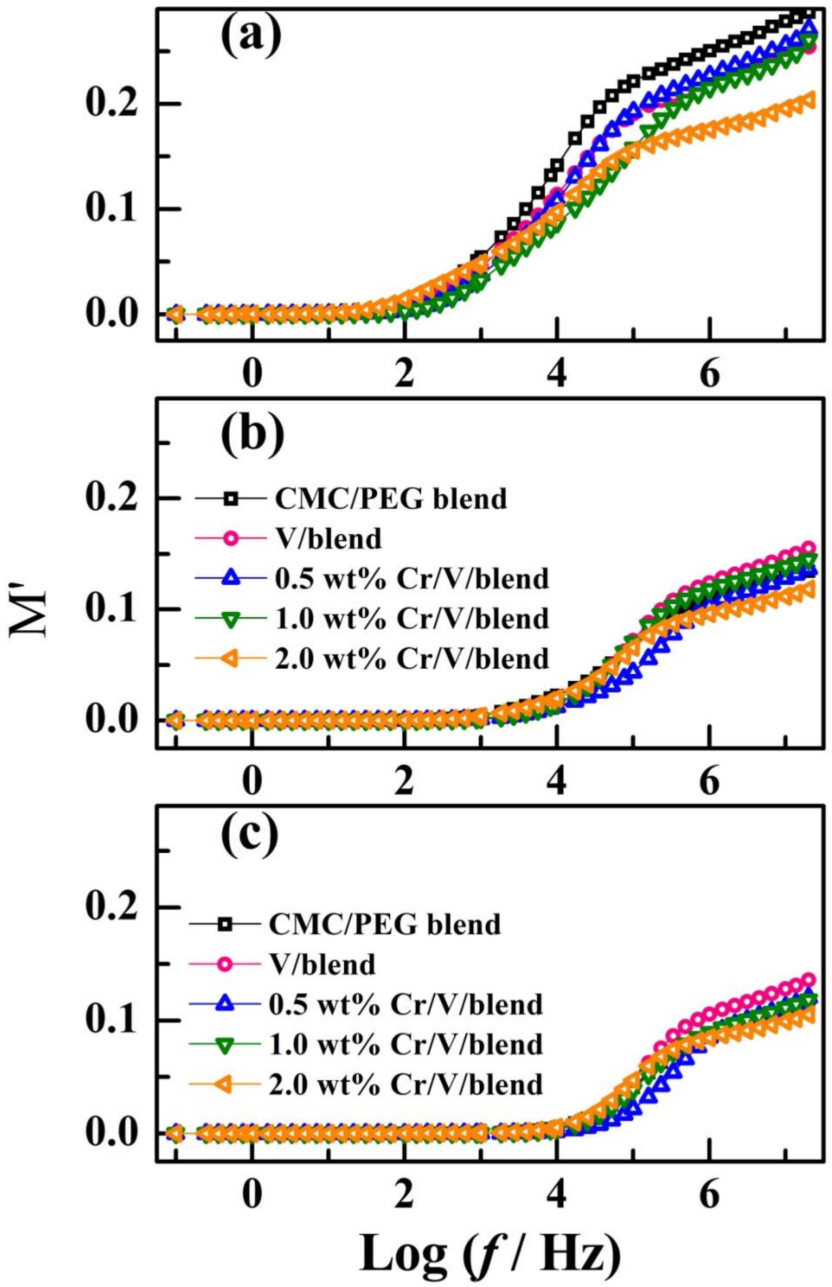

Fig. S3: The real dielectric modulus (*M′*) for CMC/PEG blend and Cr_2O_3/V_2O_5/blend.
